# Supplementary material for: “Putting the power back into community”: A mixed methods evaluation of a chronic hepatitis B training course for the Aboriginal health workforce of Australia’s Northern Territory
Source: PLoS One. 2024 Jan 24;19(1):e0288577. doi: 10.1371/journal.pone.0288577 (PMC10807824; doi:10.1371/journal.pone.0288577)
Supplement: Attachment — Submitted filename: Response to Reviewers.docx [file pone.0288577.s003.docx]

Dear Editor,

Thank you and the PLOS ONE team for your feedback and support throughout the review process. I have addressed the journal requirements below.

I also thank the two reviewers for reading and commenting on our manuscript. We are very appreciative of the feedback and feel it has strengthened the manuscript. Please see below for a detailed response to reviewers and the journal. I have attached the revised manuscript with and without tracked changes.

**Reviewers feedback**

Reviewer #1: Thank you for the opportunity to review this manuscript titled: “Putting the power back into community”: A mixed methods evaluation of a chronic hepatitis B training course for the Aboriginal health workforce of Australia’s Northern Territory. The authors must be congratulated on producing such a high quality manuscript that provides significant detail about the study, findings and context; the latter being incredibly important in teaching others how to conduct codesign methodologies successfully. I am extremely impressed by the writing, it is clear and without errors. I have only minor comments (which seems extraordinary considering that this is a long paper):

*Thank you for your generous feedback.*

L143 - I realise you provide a description of the qualifications of the Aboriginal health workforce in the acknowledgements, but I think it would be useful to briefly describe the role, including qualification and scope of practice, in the body of the paper.

*Thank you. Yes, we agree that describing this in the introduction will help the reader. I have added the following.*

*“The Aboriginal health workforce in the NT includes clinical and non-clinical roles. Aboriginal Health Practitioners (AHPs) have relevant qualifications in Aboriginal and Torres Strait Islander Primary Health Care practice, are professionally registered with the Australian Health Practitioner Regulation Agency (AHPRA) and have clinical and specialist functions. Aboriginal Community Workers (ACWs) have a range of non-clinical roles, including health promotion.”*

L182 - the scope of the Aboriginal health workforce in delivering CHB care needs to be described.

*Thank you. I have added the following.*

*“The Aboriginal health workforce is a vital connection between community members and health services and act as a patient advocate. Their role in CHB care, management and prevention is varied and includes screening - informing people of the need for a test and performing venepuncture, diagnosis - explaining to a person in their preferred language, management - explaining the importance of monitoring and care and supporting people to come into clinic, contact tracing - understanding family, kinship and household connections, vaccination; health promotion and reducing shame and stigma through improving health literacy and education.”*

L231 - Who conducted the interviews after the training? Were the facilitators also the evaluators? This needs to be explained and acknowledged in the limitations or explained.

*Thank you, this is an important point. I have added this to the methods and to the discussion in the limitations.*

*"A mix of facilitators and research staff conducted interviews”*.

*“The semi-structured interviews were conducted by Aboriginal and Torres Strait Islander and non-Indigenous facilitators and research staff. We acknowledge that having facilitators as interviewers may have influenced the responses given. Conversely, our Aboriginal and Torres Strait Islander research and teaching team also thought this might act as an enabler as “we may get more accurate answers when there is a connection and trust”.*

L243 - minor spelling mistake - "interviews" not "interviewed".

*Thank you. I have corrected it to “interviews”.*

L556 - insert "a" - "...is known to be [a] major barrier..."

*Thank you, “a” is now inserted.*

This manuscript is a joy to read because it is well written and generous in sharing all the insights and tips gathered through conducting such an extensive project. That you have demonstrated an improvement in knowledge and attitudes through delivery of this course and have expanded the CHB workforce in the Northern Territory is remarkable. Congratulations.

*Thank you, very much appreciated.*

Reviewer #2: “Putting the power back into community”: A mixed methods evaluation of a chronic
hepatitis B training course for the Aboriginal health workforce of Australia’s Northern
Territory

Introduction
Clear, comprehensive
Might have been good to detail which Aboriginal languages were included in the team identity and the participants.
Would have been good to outline Freirian principles of pedagogy.

*Thank you.*

*I have now included the language and cultural groups of the Aboriginal and Torres Strait Islander teaching and research team in materials and methods, in researcher reflexivity.*

*“TDS, a Tiwi woman, has been an Aboriginal Health Practitioner (AHP) for 19 years. PMW, AHP and artist is a Ngangi speaker and lives in Nauiyu community on Malak Malak land. SW, a Warnidilyakwa woman and Anindilyakwa speaker from Groote Eylandt has worked as an AHP for over 20 years. SN, a Gurindji woman and AHP for 24 years. GG is an Aboriginal Community Worker (ACW), researcher, proud Yolŋu man, and Yolŋu Matha speaker. SB, TN, and AD are proud Yolŋu women and senior AHPs and Yolŋu Matha speakers. LB, an AHP for over 40 years, grew up in West Arnhem Land with Iwaidja speaking and mainland Kunwinku clans. CR is a proud Arrernte, Kaytete woman and has extensive family and kinship relationships across the NT. KSC is a proud Arrernte and Anmatjere woman and senior AHP.”*

*I have added the participants languages in the results.*

*“The rich language diversity of the NT was reflected with over 20 distinct first languages spoken by the participants, including Burarra, Kunwinjku, Ndjebbana, Yolŋu Matha, Tiwi, Ngangi, Anindilyakwa, Gurindji, Murrinh Patha, Arrernte, Kriol, Pitjantjatjara, Walpiri, Kaytetye, Nunggubuyu, Luritja, Warumungu, Anmatyerr and English.”*

*I have now outlined Freirean pedagogy in the introduction with the following.*

“*Freirean pedagogy is a progressive educational approach rooted in the principles of critical thinking, social justice, and empowerment (ref). It involves praxis, an iterative cycle of awareness, and encompasses action with reflection and reflection with action (ref).*

Methods
Appropriate
Would have been good in “Reflexivity” to mention specific cultural background was represented amongst the Aboriginal member of the team

*Thank you. We are delighted to add more detail. We had reduced due to the length of the manuscript. In consultation with the Aboriginal research and teaching team, we have included.*

*“TDS, a Tiwi woman, has been an Aboriginal Health Practitioner (AHP) for 19 years. PMW, AHP and artist, is a Ngangi speaker and lives in Nauiyu community, on Malak Malak land. SW, a Warnidilyakwa woman and Anindilyakwa speaker from Groote Eylandt, has worked as an AHP for over 20 years. SN, a Gurindji woman and AHP for 24 years. GG is an Aboriginal Community Worker (ACW), researcher and proud Yolŋu man and Yolŋu Matha speaker. SB, TN, and AD are proud Yolŋu women and senior AHPs and Yolŋu Matha speakers. LB, an AHP for over 40 years, grew up in West Arnhem Land with Iwaidja speaking and mainland Kunwinku clans. CR is a is a proud Arrernte, Kaytete woman and has extensive family and kinship relationships across the NT. KS is a proud Arrernte and Anmatjere woman and senior AHP.”*

Results
Clear, concise, accurate
Would have been good to list Aboriginal languages spoken amongst Aboriginal Liaison languages spoken.

*Thank you for this suggestion. I have included the following in the results:*

*“The rich language diversity of the NT was reflected with over 20 distinct first languages spoken by the participants, including Burarra, Kunwinjku, Ndjebbana, Yolŋu Matha, Tiwi, Ngangi, Anindilyakwa, Gurindji, Murrinh Patha, Arrernte, Kriol, Pitjantjatjara, Walpiri, Kaytetye, Nunggubuyu, Luritja, Warumungu, Anmatyerr and English.”*

Discussion
Very comprehensive

*Thank you, very much appreciated.*

**Journal Requirements:**

1. Please ensure that your manuscript meets PLOS ONE's style requirements, including those for file naming. The PLOS ONE style templates can be found at 
<https://journals.plos.org/plosone/s/file?id=wjVg/PLOSOne_formatting_sample_main_body.pdf> and 
<https://journals.plos.org/plosone/s/file?id=ba62/PLOSOne_formatting_sample_title_authors_affiliations.pdf>

*Thank you, I have made the necessary adjustments to the font, headings, and format*.

2. Please include a complete copy of PLOS’ questionnaire on inclusivity in global research in your revised manuscript. Our policy for research in this area aims to improve transparency in the reporting of research performed outside of researchers’ own country or community. The policy applies to researchers who have travelled to a different country to conduct research, research with Indigenous populations or their lands, and research on cultural artefacts. The questionnaire can also be requested at the journal’s discretion for any other submissions, even if these conditions are not met.  Please find more information on the policy and a link to download a blank copy of the questionnaire here: <https://journals.plos.org/plosone/s/best-practices-in-research-reporting>. Please upload a completed version of your questionnaire as Supporting Information when you resubmit your manuscript.

*Thank you. I have completed and uploaded the “Inclusivity in global research form “with the revised manuscript.*

*I have included the following in the methods section.*

***Inclusivity in global research***

*“Additional information regarding the ethical, cultural, and scientific considerations specific to inclusivity in global research is included in the Supporting Information (S1 Checklist).”*

3. Note from Emily Chenette, Editor in Chief of PLOS ONE, and Iain Hrynaszkiewicz, Director of Open Research Solutions at PLOS: Did you know that depositing data in a repository is associated with up to a 25% citation advantage (<https://doi.org/10.1371/journal.pone.0230416>)? If you’ve not already done so, consider depositing your raw data in a repository to ensure your work is read, appreciated and cited by the largest possible audience. You’ll also earn an Accessible Data icon on your published paper if you deposit your data in any participating repository (<https://plos.org/open-science/open-data/#accessible-data>).

*Thank you. Our ethics does not allow this.*

4. Thank you for stating in your Funding Statement: 
"This research is part of the Hep B PAST project, which receives a National Health and Medical Research Council (NHMRC) partnership grant, GNT1151837. <https://www.nhmrc.gov.au/>

KH is undertaking a PhD and has an NHRMC scholarship, GNT1190918."

Please provide an amended statement that declares *all* the funding or sources of support (whether external or internal to your organization) received during this study, as detailed online in our guide for authors at <http://journals.plos.org/plosone/s/submit-now>.  Please also include the statement “There was no additional external funding received for this study.” in your updated Funding Statement. 
Please include your amended Funding Statement within your cover letter. We will change the online submission form on your behalf.

*Thank you for amending the Funding Statement in the online submission form. I have included the below in the cover letter.*

*“The NHMRC supported this work. This research is part of the Hep B PAST project, which receives an NHMRC partnership grant, GNT1151837. KH is undertaking a PhD and has an NHRMC scholarship, GNT1190918.* *Funders played no role in the study design, analysis or the decision to publish. There was no additional external funding received for this study.”*

*I have also added “There was no additional external funding received for this study” to the manuscript.*

5. In your Data Availability statement, you have not specified where the minimal data set underlying the results described in your manuscript can be found. PLOS defines a study's minimal data set as the underlying data used to reach the conclusions drawn in the manuscript and any additional data required to replicate the reported study findings in their entirety. All PLOS journals require that the minimal data set be made fully available. For more information about our data policy, please see <http://journals.plos.org/plosone/s/data-availability>.

Upon re-submitting your revised manuscript, please upload your study’s minimal underlying data set as either Supporting Information files or to a stable, public repository and include the relevant URLs, DOIs, or accession numbers within your revised cover letter. For a list of acceptable repositories, please see <http://journals.plos.org/plosone/s/data-availability#loc-recommended-repositories>. Any potentially identifying patient information must be fully anonymized.

Important: If there are ethical or legal restrictions to sharing your data publicly, please explain these restrictions in detail. Please see our guidelines for more information on what we consider unacceptable restrictions to publicly sharing data: <http://journals.plos.org/plosone/s/data-availability#loc-unacceptable-data-access-restrictions>. Note that it is not acceptable for the authors to be the sole named individuals responsible for ensuring data access.

We will update your Data Availability statement to reflect the information you provide in your cover letter.

*Thank you could you please amend our Data Availability statement to reflect that there are ethical restrictions to sharing our data publicly. Please see below, which I have added to the cover letter.*

*I have added the following to the cover letter.*

*“**There are ethical restrictions to sharing our data publicly. The ethics approval specifies, “No third party will be given access to or copies of the data”, Human Research Ethics Committee of the Northern Territory Department of Health and Menzies School of Health Research - HREC 15-2417. There were additional privacy concerns, from within the Aboriginal and Torres Strait Islander author group and from the Aboriginal and Torres Strait Islander communities involved in Hep B PAST, of sharing de-identified data, as it was perceived that the potential for re-identification is greater given the small population and workforce size. Data may be available for reasonable requests through the Hep B PAST steering committee, email:* [*Hepbpast@menzies.edu.au*](mailto:Hepbpast@menzies.edu.au)*. Phone +61889468687 or* [*kelly.hosking@menzies.edu.au*](mailto:kelly.hosking@menzies.edu.au) *Phone +61 472817647 as the corresponding author.*

*Thank you for updating the Data Availability statement to reflect the information provided in my cover letter.*

6. We note that you have stated that you will provide repository information for your data at acceptance. Should your manuscript be accepted for publication, we will hold it until you provide the relevant accession numbers or DOIs necessary to access your data. If you wish to make changes to your Data Availability statement, please describe these changes in your cover letter and we will update your Data Availability statement to reflect the information you provide.

*Thank you, and I apologise, I must have incorrectly selected that option.*

*Please amend our Data Availability statement to reflect that there are ethical restrictions to sharing our data publicly. Please see above, which I have added to the cover letter. Please refer to the cover letter*

*Data may be available for reasonable requests through to the Hep B PAST steering committee, email:* [*Hepbpast@menzies.edu.au*](mailto:Hepbpast@menzies.edu.au)*. Phone +61889468687 or* [*kelly.hosking@menzies.edu.au*](mailto:kelly.hosking@menzies.edu.au)*.*

7. One of the noted authors is a group or consortium [Hep B PAST partnership]. In addition to naming the author group, please list the individual authors and affiliations within this group in the acknowledgements section of your manuscript. Please also indicate clearly a lead author for this group along with a contact email address.

*Thank you, I have added to the acknowledgements section of the manuscript.*

*“We acknowledge the Hep B PAST partnership. See Supplementary Information (S2).”*

*A/Prof Jane Davies is the Chief Investigator for Hep B PAST and can be contacted at* [*jane.davies@menzies.edu.au*](mailto:jane.davies@menzies.edu.au)*. Kelly Hosking is this manuscript's lead and corresponding author and can be contacted at* [*kelly.hosking@menzies.edu.au*](mailto:kelly.hosking@menzies.edu.au)*.*

*I have uploaded the members of the consortium/partnership within the manuscript as S2.*

*When re-reviewing the authorship requirement, I believe one of the people named as an acknowledgement, Cheryl Ross, meets the authorship criteria. I have added her to the authorship list. Can PLOS ONE please update the online submission to reflect this, if required? Cheryl has read the manuscript and consents to it being submitted/published. Her affiliation is Menzies School of Health Research. Her contact details are:* [*Cheryl.ross@menzies.edu.au*](mailto:Cheryl.ross@menzies.edu.au)

8. We note that Figure 1 in your submission contain map/satellite images which may be copyrighted. All PLOS content is published under the Creative Commons Attribution License (CC BY 4.0), which means that the manuscript, images, and Supporting Information files will be freely available online, and any third party is permitted to access, download, copy, distribute, and use these materials in any way, even commercially, with proper attribution. For these reasons, we cannot publish previously copyrighted maps or satellite images created using proprietary data, such as Google software (Google Maps, Street View, and Earth). For more information, see our copyright guidelines: <http://journals.plos.org/plosone/s/licenses-and-copyright>.
 
We require you to either (1) present written permission from the copyright holder to publish these figures specifically under the CC BY 4.0 license, or (2) remove the figures from your submission:
 
a. You may seek permission from the original copyright holder of Figure 1 to publish the content specifically under the CC BY 4.0 license.  
 
We recommend that you contact the original copyright holder with the Content Permission Form (<http://journals.plos.org/plosone/s/file?id=7c09/content-permission-form.pdf>) and the following text:
“I request permission for the open-access journal PLOS ONE to publish XXX under the Creative Commons Attribution License (CCAL) CC BY 4.0 (<http://creativecommons.org/licenses/by/4.0/>). Please be aware that this license allows unrestricted use and distribution, even commercially, by third parties. Please reply and provide explicit written permission to publish XXX under a CC BY license and complete the attached form.”
 
Please upload the completed Content Permission Form or other proof of granted permissions as an ""Other"" file with your submission.
 
In the figure caption of the copyrighted figure, please include the following text: “Reprinted from [ref] under a CC BY license, with permission from [name of publisher], original copyright [original copyright year].”

*This was created by NT Health communications and media team. We have discussed with the legal team and have the correct permission to publish. The “Request for Permission to Publish Content “has been signed and uploaded as “Other”.*

b. If you are unable to obtain permission from the original copyright holder to publish these figures under the CC BY 4.0 license or if the copyright holder’s requirements are incompatible with the CC BY 4.0 license, please either i) remove the figure or ii) supply a replacement figure that complies with the CC BY 4.0 license. Please check copyright information on all replacement figures and update the figure caption with source information. If applicable, please specify in the figure caption text when a figure is similar but not identical to the original image and is therefore for illustrative purposes only.
The following resources for replacing copyrighted map figures may be helpful:
 
USGS National Map Viewer (public domain): <http://viewer.nationalmap.gov/viewer/>
The Gateway to Astronaut Photography of Earth (public domain): <http://eol.jsc.nasa.gov/sseop/clickmap/>
Maps at the CIA (public domain): <https://www.cia.gov/library/publications/the-world-factbook/index.html> and <https://www.cia.gov/library/publications/cia-maps-publications/index.html>
NASA Earth Observatory (public domain): <http://earthobservatory.nasa.gov/>
Landsat: <http://landsat.visibleearth.nasa.gov/>
USGS EROS (Earth Resources Observatory and Science (EROS) Center) (public domain): [http://eros.usgs.gov/#](http://eros.usgs.gov/)
Natural Earth (public domain): <http://www.naturalearthdata.com/>

9. Please review your reference list to ensure that it is complete and correct. If you have cited papers that have been retracted, please include the rationale for doing so in the manuscript text, or remove these references and replace them with relevant current references. Any changes to the reference list should be mentioned in the rebuttal letter that accompanies your revised manuscript. If you need to cite a retracted article, indicate the article’s retracted status in the References list and also include a citation and full reference for the retraction notice.

*One reference “38” has changed from “preprint” to published.*

*Reviewer 2 suggested adding a description of Freirean pedagogy to the introduction, which I have done. This has meant that the reference numbers have changed throughout the manuscript.*

Thank you again for reviewing our manuscript. Please let me know if you have any questions or require anything else.

Kind regards,

Kelly
